# Impact of a Pilot Project for Integrated Care on Hospitalization Rate among Older Adults in South Korea

**DOI:** 10.5334/ijic.7665

**Published:** 2024-05-28

**Authors:** Hyo Jung Bang, Ae Jung Yoo, Hyun Ji Lee, Jae Woo Choi

**Affiliations:** 1 Integrated Care Research Center, Health Insurance Research Institute, National Health Insurance Service, Gangwon, Korea

**Keywords:** integrated care, hospitalization rate, difference-in-difference, older adult

## Abstract

**Introduction:**

Since 2019, the Korean government has implemented a pilot project for integrated care to encourage healthy aging of older adults. This study investigated the changes in hospitalization rates among older adults who participated in the integrated care pilot project.

**Methods:**

Administrative survey data collected from 13 local governments and the National Health Insurance Database were used in present study. The participants comprised 17,801 older adults who participated in the pilot project between August 01, 2019 and April 30, 2022 and 68,145 matched controls. A propensity score matching method was employed to select the control group, and this study employed difference-in-differences (DID) approach to examine variations in the hospitalization rate.

**Results:**

The DID analysis revealed that the odds ratio for rates of hospitalization among older adults who participated in the pilot project was 0.88 (95% confidence interval [CI] 0.84, 0.91) in comparison to control group. In specifically, as compared to the control group, the odds ratio for hospitalization rates among the pilot project’s discharged patients was 0.17 (95% CI 0.15, 0.20). Although not statistically significant, the odds ratio of older adults who utilized LTCI services was 0.93 (95% CI 0.83, 1.05), and the odds ratio of older adults who applied for LTCI but were rejected or were intensive social care was 1.09 (95% CI 0.95, 1.26) compared to the comparison group.

**Discussion:**

The findings imply that the discharged patient group had greater medical demands than the other types, and it can be claimed that this is the group that may anticipate greater efficacy while using health services. In addition, the integrated care services provided by the pilot project have the effect of reducing unnecessary hospitalization such as social hospitalization.

**Conclusion:**

Participants in the integrated care pilot project showed a lower hospitalization rate than the older adults who did not participate in the project but had similar characteristics. In particular, the admission rate of discharged patients showed a sharp decline.

## Introduction

South Korea is aging at the fastest rate worldwide, and its average life expectancy has increased, reaching 83.6 years as of December 2021 [1]. According to the National Survey of older Koreans (2020), 54.9% of older adults has two or more chronic diseases. Taken together, this indicates that the number of older adults needing long-term treatment or care is rapidly increasing [2]. As the unhealthy older adults increases, the burden of medical expenses on them also increases.

However, it is worth noting that a significant portion of the medical expenses of the older adults are incurred by hospitalization [3]. Careful management of physical health and functional status is needed because a history of hospitalization in the older adults leads to repeated readmission, admission to a long-term care facility, and death, and hospitalization in a medical institution means deterioration of physical health and functional status [3,4]. Social hospitalization, a major cause of increased medical expenses for older adults, can be defined as a phenomenon in which the need for hospitalization is clinically low, but inappropriate-, prolonged hospitalization due to personal or socioeconomic conditions occurs [5,6,7,8]. According to research conducted in Korea, 74% of inpatients in long-term care hospitals were hospitalized by their families, not by themselves, and 6.1% of inpatients were physically impaired with low medical needs [9]. Therefore, to alleviate the increase in medical expenses and socioeconomic burden of older adults due to population aging, it is necessary to establish a system in which the older adults can effectively manage their health and receive services that meet their needs from the time they live in the community [10,11,12].

The Korean government has undertaken a pilot project for integrated care in 13 of 229 local governments since 2019 to promote healthy aging in communities where older adults live and to reduce their financial expenditures for care services. The pilot project focuses on improving the connections between healthcare services and home-based services (home visit care, home visit bathing, etc.) provided by long-term care insurance (LTCI), supported housing, and social services and expanding underdeveloped community services. In particular, the core components of Korea’s integrated care services include medical care, social welfare, long-term care, housing, and daily life support (meals, mobility, and household assistance). A detailed description of the pilot project is included in the supplementary file.

Various studies have been conducted to verify the effects of integrated care on hospital admissions worldwide; however, each has shown conflicting results. A prior study showed that various programs of integrated care providing medical and social services to the older adults who resided in their communities helped reduce hospital admissions and nursing home utilization in many countries [13,14,15,16]. In contrast, in studies conducted in Canada, integrated care did not have a statistically significant effect on hospital admission [17,18,19]. A systematic review conducted in England on the effect of integrated care services on hospital admissions found that some studies reported a positive effect, while others showed no effect or a negative effect [20]. These conflicting results are presumed to be due to differences in the social service system or service content of each pilot project. A lack of research in this area in Korea necessitates comparative research with other countries by verifying the effect of the integrated care pilot project that reflects the characteristics of Korea.

This study aimed to examine whether there was a difference in hospitalization rates between older adults who participated in a pilot project for integrated care and those who did not. In addition, because the priority target types were classified into three types according to the purpose of the pilot project, the differences in hospitalization rates were also analyzed according to the type of participant.

## Research Methods

### Data and study sample

*In this study, National Health Insurance* data and administrative survey data accumulated by 13 local governments participating in the integrated care pilot project was analyzed in connection with each other. National Health Insurance is one of the social insurances operated by the state to which all citizens of Korea must subscribe, and is managed by the National Health Insurance Corporation. The National Health Insurance data includes information on eligibility for national health insurance and long-term care insurance, and information on death, medical service use, long-term care service use, and medical and long-term care institutions [21]. Administrative survey data were collected regularly by the 13 local governments from August 2019. It included information on participants (information on socioeconomic status such as household income, housing type, type of social security insurance, availability of caregiver, etc.), pilot project registration and termination, and services provided to the participants in the pilot project for integrated care. With the help of the central government, we gathered survey data from 13 local governments and linked them to the Korean National Health Insurance Service (KNHIS) data.

The analysis was limited to older adults who participated in the pilot project for integrated care between August 2019 and April 2022. The total number of participants in the integrated care pilot project was 25,692. Among them, those whose identities could not be confirmed, such as incorrect resident registration numbers, and those who participated in the pilot project from May to August 2022, considering the analysis target period from August 2019 to April 2022, were excluded from analysis (n = 3,134). In addition, participants who were registered in the integrated care pilot project but were not connected to any services (n = 1,889) and participants under the age of 65 (n = 1,101) were excluded from the analysis. Finally, cases in which personal information was not consented for research purposes (n = 72) and cases in which pilot projects for integrated care data and national health insurance data were not linked (n = 33) were also excluded from the analysis.

**Figure 1:**
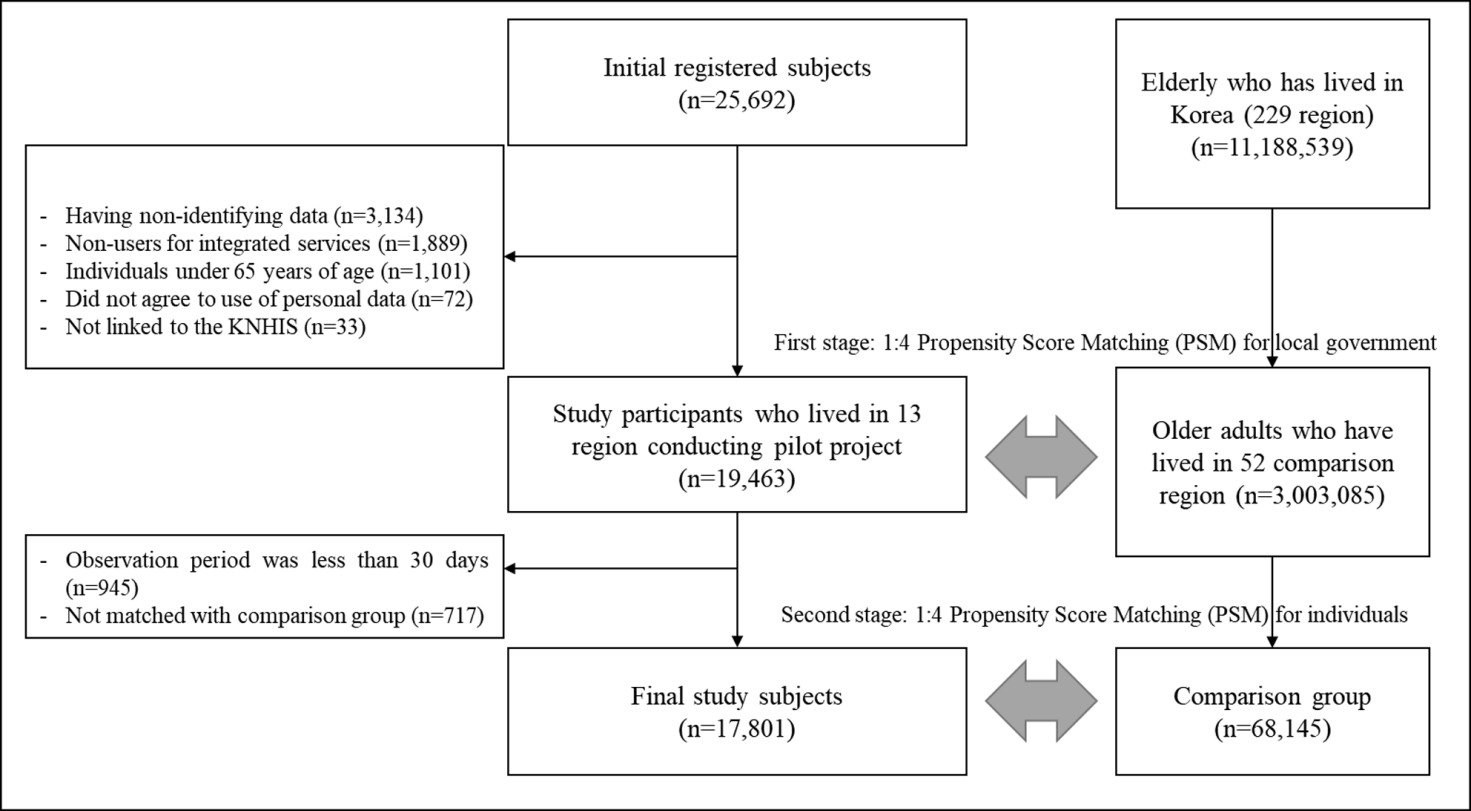
Flow chart of study participants.

To compare the effects of participation in the pilot project, we selected and analyzed a control group that lived in regions where no such pilot project was conducted. A 1:4 PSM method was used to select the control group. The propensity score matching technique can reduce the possibility of selection bias and pair individuals with the same or similar propensity scores into two different groups [22]. Because the integrated care pilot project is carried out at the regional level, and the provision of integrated services is relatively influenced by the composition of the region’s population and resources, this study used a two-stage PSM (first stage: region [local government], second stage: individual). In the first stage of PSM, this study chose 52 comparison regions that shared four characteristics with the 13 local governments: percentage of the older adults, total population, neighborhood deprivation index [23], and region type. In the second stage of PSM, participants with similar individual characteristics (sex, age, household income, Charlson Comorbidity Index (CCI), inpatient history, psychiatric diseases, disability, LTCI grade, and living status) were extracted and matched with older adults who reside in each of the four matched regions. To strengthen the trustworthiness of the results, we further removed cases (n = 945) in which the observation time was less than 30 days following the date of registration. Research participants (n = 717) who were not matched with the comparison group were eliminated after the two-stage PSM. Through this series of processes, 17,801 people (experimental group) were finally selected for analysis, and 68,145 people were selected as a control group for comparative analysis (Figure 1).

In the pilot project, the three types of priority targets for management were separately classified and the ultimate goal achievement level of the project was managed. These were people who received long-term care services in a region other than a facility, after obtaining a service target level by meeting certain criteria set by LTCI [24]; 2,226 out of 17,801 people in the experimental group fell into this category. The second type was the experimental group, which included older adults who applied for LTCI but were not judged because the minimum level of requirements was not met and older adults who belonged to the intensive social care group of the national customized care service. The Korean government encourages the provision of intensive social care for frail older adults who require daily living assistance but are in a higher functional position than recipients of LTCI and are thus unable to receive LTCI benefits. Safety, social engagement, education, and housekeeping aid are all included in the older adults ‘s intensive social care services. Intensive social care and general care are the two service groups that the aged tailored care service targets. The intensive social care group is made up of older persons who require more daily life assistance owing to physical function restrictions than the general group; 1,675 of the experimental group were classified into the second type. The third type was discharged patients who had a history of being discharged from a medical institution within one year prior to participating in the pilot project (n = 1,895). In this study, the effects on all participants were compared and analyzed between the experimental and control groups, and each type was analyzed to classify the effects on priority participants.

### Measurement

In this study, a comparison group was selected using PSM and the comparison group with the most similar characteristics was selected by considering factors related to the outcome variables. Using data from the year of registration, household income level was determined and classified as: Q1 (20th percentile, lowest), Q2 (21st–40th percentile), Q3 (41st–60th percentile), Q4 (61st–80th percentile), and Q5 (81st–100th percentile, highest). The Charlson Comorbidity Index, calculated using records from the year prior to the pilot project’s registration date, is the total weighted score assigned to several significant medical problems [25]. The applicants’ physical functional status, physical or mental health state, and care needs were graded on a scale of 1 to 5 by the LTCI. The first major condition for which long-term facility care is advised is 1–2 Grade. Home-based care is advised for conditions like 3–5 Grade and cognitive support grades, which are less problematic than 1–2 Grade. In terms of LTCI grades, LTCI covers all LTC requirements for seniors 65 years of age and over, as well as age-related LTC needs (such as dementia) for younger adults if they become eligible through an LTC approval procedure. An LTC needs assessment team visits the applicant’s home to evaluate the applicant’s physical and cognitive abilities. The five dimensions of this 52-item instrument include physical functioning, cognitive, behavioral issues, desire for nursing care, and demand for rehabilitation. The evaluation outcomes are combined and transformed into an LTC acceptance score that ranges from 0 to 100. An applicant’s LTC grade is calculated using the LTC approval score and the LTC eligibility committee’s judgment. There are six grades in total: grades 1 (95–100 points), 2, 75–94 points, 3, 60–74 points, 4, 51–59 points, 5, and cognitive assistance grade (under 45 points and dementia) [34]. In this study, LTCI variable was divided into three categories: recipients of the intervention (grades 1–2), those who received it (grades 3–5), and those who did not (grade non-recipients). Utilizing hospitalization data from medical facilities one year before the pilot project’s registration date, the history of hospital admission was calculated. Medical records of mental illness with the International Statistical Classification of Diseases and related health problems-10 codes F00–F99 for three years prior to the pilot project’s registration date were used to measure psychiatric disorders. The number of people living in a household at the time the registration date was taken into account in this study to evaluate living alone.

### Statistical analysis

Sociodemographic, health, and physical function characteristics of the participants were analyzed using Pearson’s chi-square test and an independent t-test. Categorical variables are shown as numbers and percentages, and continuous variables as means and standard deviations. The standardized difference (SD) was used to calculate the homogeneity test for matching variables between the pilot project participants and the comparison group. In a propensity score-matched sample, SD examines the balance in baseline variables between treated and untreated participants by comparing the difference in means expressed in units of the pooled standard deviation [26]. According to some researchers, a considerable imbalance in the baseline covariate is indicated by an absolute value greater than 0.1, although there is no apparent agreement on this threshold [27].

This study used difference-in-differences (DID) methodology to examine the differences in inpatient service use rates between participants who took part in the pilot project and the comparison group. When a policy is implemented, the DID methodology, a standard tool for policy assessment, examines the independent impact of the policy’s introduction on a case group compared to a control group. Previous studies used the DID approach to estimate the impact of policy changes or the introduction of new systems [28]. The post-observation period for the study participants was established as the observation period beginning on the date of enrolment and ending on July 31, 2022. The observation period was designated from the date of participation until the pilot project’s services were no longer being used or the participant passed away. The post-observation period was given the same designation as previously based on the registration date in order to conduct the same before-after comparison. The same participation date and pre- and post-observation periods were assigned to each matched control group to track changes over the course of the same observation period.

It is vital to consider the correlation within participants because this study compared the pre-post comparison of the same participant. This study used McNemar’s test for categorical data to determine the statistical significance of the differences in the pre- and post-results of outcomes by participation in the pilot project. The generalized estimate equation method, which was used for DID analysis, can be examined by considering the peculiarities of repeated measurement data. The value of the interaction term between the variables for the pilot project participants and the time before and after the pilot project was used to calculate the outcomes in DID.

SAS 9.4 was used for all data extraction and statistical analyses (SAS Institute Inc. Cary, NC). This study was approved by the Institutional Review Board of the National Health Insurance Service (approval number: 2022-HR-03-024).

## Results

This study comprised 17,801 older adults (20.7%) who participated in the pilot project for integrated care as the experimental group, while 68,145 older adults who did not participate in the pilot project (79.3%) formed the control group. The average observation period for all participants was 652.4 days (326.2 days each before and after the lead project participation date).

Table 1 presents the general characteristics of participants of the pilot project. Among them, the proportion of women was 70.1%, which was higher than that of men, and the average age of all participants was 79.8 years. Among the participants of the pilot project, the proportion of participants with a high household income was the highest (29.8 %), and 1–2 grade of long-term care insurance accounted for 1.3%, and 3–5 grade and the cognitive support grade accounted for 15.9%. In addition, 44.0% had a history of hospitalization in a medical institution within one year, 29.5% had a disability, and 41.1% had a history of mental illness. Participants with a CCI of 1 or higher, indicating medical severity, accounted for 89.2%, and 57.4% of the participants lived alone. No variables showed statistically significant differences among the common characteristics used in this study.

**Table 1:** General characteristics of study subjects.


VARIABLES		PRE-PSM				SD	POST-PSM		SD
	
		OLDER ADULTS WHO PARTICIPATED IN PILOT PROJECT		OLDER ADULTS WHO DID NOT PARTICIPATED IN PILOT PROJECT			OLDER ADULTS WHO DID NOT PARTICIPATED IN PILOT PROJECT		
		
		N	%	N	%		N	%	

Total		17,801	20.7	3,003,085	100.0		68,145	79.3	

Sex						0.314			–0.037

	Men	5,318	29.9	1,347,429	44.9		19,218	28.2	

	Women	12,483	70.1	1,655,656	55.1		48,927	71.8	

Age (mean ± standard deviation)		79.8	6.6	72.6	7.7	1.008	79.9	6.8	–0.004

Household Income						0.314			–0.037

	Q1 (Lowest)	5,039	28.3	172,117	5.7		18,435	27.1	

	Q2	2,787	15.7	563,815	18.8		10,468	15.4	

	Q3	2,253	12.7	489,602	16.3		8,425	12.4	

	Q4	2,422	13.6	651,630	21.7		9,169	13.5	

	Q5 (Highest)	5,300	29.8	1,082,021	36.0		21,648	31.8	

Grade of long-term care insurance						0.385			0.055

	1–2 grade	224	1.3	50,986	1.7		686	1.0	

	3–5 grade and cognition support grade	2,830	15.9	197,005	6.6		9,684	14.2	

	None	14,747	82.8	2,755,094	91.7		57,775	84.8	

History of Inpatients						0.445			0.030

	Yes	7,833	44.0	704,234	23.5		28,965	42.5	

	No	9,968	56.0	2,298,851	76.5		39,180	57.5	

Disability						0.385			0.055

	Yes	5,259	29.5	418,805	13.9		18,443	27.1	

	No	12,542	70.5	2,584,280	86.1		49,702	72.9	

Psychiatric diseases						0.445			0.023

	Yes	7,312	41.1	630,002	21.0		27,210	39.9	

	No	10,489	58.9	2,373,083	79.0		40,935	60.1	

CCI						0.314			–0.037

	0	1,917	10.8	850,742	28.3		7,198	10.6	

	1	2,924	16.4	698,596	23.3		11,634	17.1	

	2	2,999	16.8	526,164	17.5		11,925	17.5	

	3	2,731	15.3	356,147	11.9		10,801	15.9	

	4	2,304	12.9	230,398	7.7		8,758	12.9	

	5	1,739	9.8	141,107	4.7		6,353	9.3	

	6≤	3,187	17.9	199,931	6.7		11,476	16.8	

Living alone						0.706			0.001

	Yes	10,215	57.4	736,471	24.5		39,104	57.4	

	No	7,586	42.6	2,251,200	75.0		29,041	42.6	


Abbreviation. PSM: propensity score matching, SD: standardized difference, CCI: Charlson’s comorbidity index.

Table 2 shows the changes in hospitalization rates in medical institutions for the experimental and control participants. The rate of hospitalization at a medical institution for the experimental group was 39.4% before participating in the project, and decreased by 5.2% to 34.2% post participation. However, in the control group, it decreased by 1.8% from 30.7% to 28.9% during the same period. The DID analysis revealed that the odds ratio [OR] for rates of hospitalization among older adults who participated in the pilot project was 0.88 (95% confidence interval [CI] 0.84, 0.91) in comparison to control group.

**Table 2:** The results for changes of hospital admission rates by participation of pilot project for integrated care.


SUBJECTS	N	PRE-REGISTRATION		POST-REGISTRATION		PRE-POST DIFFERENCES		DID	
			
		N	%	N	%	%P	P-VALUE	OR (95% CI)	P-VALUE

Older adults who participated in pilot project	17,801	7,006	39.4	6,091	34.2	–5.2	<.001	0.88 (0.84–0.91)	<.001

Comparison group	68,145	20,940	30.7	19,676	28.9	–1.8	<.001		


Abbreviation. LTCI: long-term care insurance, STD: standard deviation, DID: difference-in-differences.

Table 3 shows the results of analyzing the change in hospital admission rate by classifying the priority management groups by type. First, in the case of long-term home care recipients, the number of participants (n = 2,226) who participated in the pilot project for integrated care decreased by 1.7%, from 40.9% before participating in the project to 39.2% after participating in the project, however, this was not statistically significant. In the control group (n = 8,181), there was no change in the hospitalization rate in medical institutions before and after participating in the project. The results of the DID analysis of hospital admission rates between the two groups did not show a statistically significant difference (OR 0.93, 95% CI 0.83, 1.05). In the second subtype, the intensive social care group for those who participated in the pilot project (n = 1,675), the rate of admission to medical institutions was 34.3% before participation in the project, and decreased by 1.3% to 33.0% post participation; however, the difference was not statistically significant. Conversely, the control group (n = 6,400) showed a 2.6% decrease in hospitalization rate, from 30.6% before participating in the project to 28.0% after participating in the project. The results of the DID analysis between the two groups were also not statistically significant (OR 1.09, 95% CI 0.95, 1.26).

**Table 3:** The results for changes of hospital admission rates by subgroups in pilot project for integrated care.


TYPE	SUBJECTS	N	PRE-REGISTRATION		POST-REGISTRATION		PRE-POST DIFFERENCES		DID	
			
			N	%	N	%	%P	P-VALUE	OR (95% CI)	P-VALUE

Older adults who utilized LTCI services	Older adults who participated in pilot project	2,226	910	40.9	872	39.2	–1.7	0.179	0.93 (0.83–1.05)	0.238

	Comparison group	8,181	3,081	37.7	3,063	37.4	–0.3	0.726		

Older adults who applied for LTCI but were rejected or were intensive care group of customized care services for older adults	Older adults who participated in pilot project	1,675	575	34.3	553	33.0	–1.3	0.362	1.09 (0.95–1.26)	0.229

	Comparison group	6,400	1,961	30.6	1,791	28.0	–2.6	<.001		

Patients who discharged from medical institution	Older adults who participated in pilot project	1,895	1,668	88.0	876	46.2	–41.8	<.001	0.17 (0.15–0.20)	<.001

	Comparison group	7,175	2,972	41.4	2,317	32.3	–9.1	<.001		


Abbreviation. LTCI: long-term care insurance, STD: standard deviation, DID: difference-in-differences.

Finally, for the type of patients discharged from medical institutions, 88.0% of those who participated in the pilot project (n = 1,895) were hospitalized at a medical institution before participating in the project. However, post participation, the admission rate to medical institutions decreased to 46.2%; in the control group (n = 7,175), it decreased from 41.4%, to 32.3%. As a result of the DID analysis, as compared to the control group, the odds ratio for hospitalization rates of discharged patients was 0.17 (95% CI 0.15, 0.20). Among the priority management groups participating in the pilot project, the difference in hospitalization rates between the experimental and control groups were statistically significant only for patients discharged from medical institutions.

## Discussion

This study evaluated differences in hospitalization between older adults who participated in a pilot project for integrated care in Korea and those who did not. Differences in hospitalization rates were confirmed by classifying the three priority management target types set in the pilot project for integrated care. The results of the DID analysis showed that the odds ratio of the hospitalization rate decreased significantly for the older adults who participated in the integrated care pilot project. In addition, looking at the detailed results of the three priority management target types set in the pilot project for integrated care, the rate of hospitalization decreased in all types, but the rate for LTCI beneficiaries and the older adults who applied for LTCI but were rejected, or the intensive social care group of customized care services were not statistically significant. LTCI beneficiaries and the older adults who applied for LTCI but were rejected, or the intensive social care group are older adults who were receiving care services from existing systems such as long-term care insurance or customized care services. Therefore, even if integrated care provided complementary services to these types of participants, the effect would not have been greater than that of discharged patients. Although it was not statistically significant in the above two types, the ultimate purpose of integrated care and medical support is not simply to reduce costs or hospitalization rates, but to maintain a dignified life for individuals. Therefore, in future research, it is necessary to analyze the two types that were not statistically significant by considering additional dependent variables such as change in subjective quality of life, level of dignity, and change in burden of care.

The hospitalization of all the older adults who participated in the pilot project for integrated care was reduced, and the same services were provided to the three groups that needed priority management, which was effective in all groups; however, the admission rate of the discharged patient type showed a particularly high decrease. This finding can be interpreted from two perspectives. First, the discharged patient group had a higher risk of hospitalization than the other groups. The analysis showed that the rate of admission before participating in the project for the discharged patient group was 88.0%, which was significantly higher than that for the other groups. This means that the discharged patient group had higher medical needs than the other types, and it can be said that this is the group that can expect higher effectiveness than other types when intervening in health services. In South Korea, the healthcare delivery system is not adequately established, and the regional-based health management system, including the primary care physician system, is deficient. Therefore, there are many cases in which patients discharged from medical institutions use hospital services again rather than using primary clinics in the region. In the pilot project for integrated care, visiting medical services based on the medical needs of the participants, customized exercise education, nutritional education, lunch box support services, and medication guide services were provided. These services help discharged patients with relatively high medical needs receive sufficient health and social care while living in the region without being hospitalized in a medical institution and prevent the deterioration of their health. The significant decrease in the hospitalization rate of the discharged patients after participating in the project is evidence of such an effect. In a prior study, it was shown that the Program of All-Inclusive Care for the Elderly, which provides full medical care to the older adults who reside in their communities, helped to reduce hospital and nursing home utilization in the United States [19,29].

Second, the decrease in the older adults ‘s admission to medical institutions through participation in the pilot project can be interpreted as not only improving their physical health and functional status but also reducing the use of unnecessary medical institutions. Social hospitalization accounts for a large proportion of unnecessary hospitalizations. A phenomenon known as “social hospitalization” occurs when a patient receives improper or prolonged care due to personal or economic reasons even when there is little clinical justification for their admission [30,31]. Social hospitalization with relatively low medical needs is likely to result in repeated hospitalizations after discharge because the ultimate cause, socioeconomic needs, are not resolved, even if a certain amount of treatment is received at a medical institution. In the pilot project for integrated care, not only were medical and health management services provided to participants, but services corresponding to complex needs, such as residential environment improvement, welfare services, and economic support, were also provided. These multidimensional services and support helped reduce unnecessary hospitalizations in the discharged patient group. Previous studies also showed that unnecessary hospitalization was reduced by providing integrated care, including home nursing, housing and economic support, and nutrition management to older adults living at home [15,32,33]. In this way, if social hospitalization is reduced through various integrated care services, not only can the consumption of health insurance funds due to unnecessary repeated hospitalizations be prevented, but the quality of life of older adults with complex needs can also be expected to improve.

Our study has several limitations. First, integrated services were supplied in accordance with participants’ needs under the integrated care pilot project in Korea. As each participant received varied types and quantities of services, it was challenging to determine which particular service or set of services was responsible for the success of the pilot project. Evaluation through the development of a standardized service model will be required in the future. Second, a variety of potential confounding factors such as local resources, which may have impacted the results of this study, were not considered (the number of hospitals or long-term care institutions). Further studies that consider more factors related to the outcomes are required. In particular, more detailed analysis will need to be conducted in the future to clearly identify the causes of social hospitalizations. Through this, it is expected that the interpretation of social hospitalization explained in the results of this study will become clearer. Finally, because the pilot project for integrated care was the first of a kind in Korea, some older adults who needed the services could not have been included in the recruitment stage because of lack of knowledge. As a result, it’s possible that not all older adults with decreased mobility who require therapeutic evaluation will benefit from the findings of this study.

## Conclusion

This study is the first to explore changes in hospitalization as an effect of a pilot project on the integrated care of older adults in South Korea. The results of the DID analysis showed that the hospitalization rate decreased significantly (12%) among older adults who participated in the pilot project for integrated care compared to those who did not. In addition, for older adults’s patients who were discharged from the medical institutions, the hospitalization rate of those who participated in the pilot project for integrated care was significantly reduced by 83% compared to that of the control group. The initial pilot project, which was the subject of this study, is nearing completion, and a new project is ready to be launched in the second half of 2023. Based on the evaluation findings of the initial pilot projects, it is essential that future pilots clearly define their objectives, participant targets, and standard services. National and local governments must also adequately extend vital services such as visiting medical services in the community and maintain strong connections with Korea’s current healthcare infrastructure. In particular, the new pilot project is expected to enhance hospitalization management of older adults as it plans to strengthen healthcare services and focus on discharged patient management.

In addition, from an international perspective, hospitalization in medical institutions is known to be a main risk factor affecting repeated readmissions, long-term care facility admission, and death. Therefore, many countries are already trying to reduce hospitalization rates in various ways. Among them, providing older adults with the integrated care services they need, such as medical care and long-term care, in their local communities is an effective attempt to prevent hospitalization or readmission to medical institutions, and many countries are already implementing such policies. In this global trend, the contents of Korea’s integrated care pilot project for older adults introduced in this study and the analysis results on hospitalization can be used as meaningful evidence.

## Additional File

The additional file for this article can be found as follows:

10.5334/ijic.7665.s1 Supplementary file. Detailed description of integrated care in Korea.

## Data Accessibility Statement

Data was obtained from a third party and are not publicly available.
