# Supplementary material for: Impact of a Pilot Project for Integrated Care on Hospitalization Rate among Older Adults in South Korea
Source: Int J Integr Care. 2024 May 28;24(2):20. doi: 10.5334/ijic.7665 (PMC11141512; doi:10.5334/ijic.7665)
Supplement: Supplementary file. — Detailed description of integrated care in Korea. [file ijic-24-2-7665-s1.pdf]

Illustration of core components and support system of Korea's integrated care services

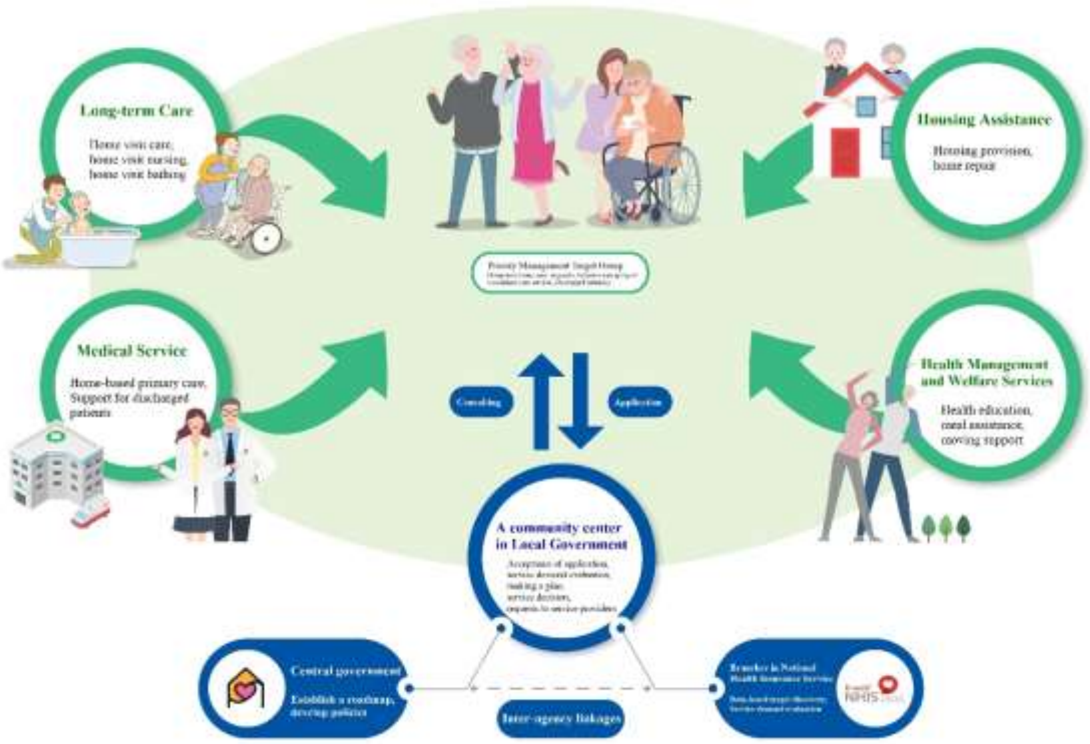

A detailed description of the reform and a pilot project

Korea executed LTCI from July 2008 in arrange to move forward the quality of life among more seasoned grown-ups encountering troubles in overseeing their day by day lives and to diminish the burden of casual family caregivers with home-based or institutional-based long-term care. People matured 65 or more seasoned can apply for the LTCI program; in the event that decided to meet the qualification criteria, based on physical working, cognition, behavioral issues, and request for nursing and recovery, recipients are neat to get home-based or institutional-based care. Domestic care administrations incorporate domestic visit care, domestic visit nursing, domestic visit washing, day and night care, short-term care, and arrangement of assistive gadgets. In the meantime, Korean government advanced the elderly customized care administrations for powerless more seasoned grown-ups who cannot be utilized with LTCI due to having generally way better useful status than LTCI recipients in show disdain toward of confronting challenges in day by day errands. The elderly customized care administrations comprise of help for security, social interest, instruction, and housekeeping.

Be that as it may, feedback has been persistently raised that it is troublesome to meet the wants of the older adults for proceeded home within the own' domestic with as it were single care program such as LTCI or elderly customized care administrations. In Korea, the care administrations of LTCI and elderly customized care administrations were inadequately, and

other vital administrations such as healthcare administrations and lodging administrations were worked in a divided way. The more seasoned grown-ups had to apply at different counters in arrange to utilize the vital administrations, and a few more seasoned individuals were not given with the essential administrations due to diverse application capabilities of care administrations. Eventually, in spite of the fact that 56.5% of community-dwelling more seasoned grown-ups in Korea need to proceed living in their claim homes indeed in case they have constrained versatility, numerous more seasoned grown-ups live and get care in private long-term care (LTC) offices or gaining strength healing centers or maybe than in their possess homes since of division and the need of care administrations in social protections and tax-based community welfare frameworks.

To support healthy aging and provide integrated necessary care services for older adults, the Korean government has implemented a pilot project for community care for older adults at 13 of the 229 local governments since June 2019. The pilot project focuses on strengthening the linkage between NHI services (e.g., inpatients, outpatients, and drug prescription) and LTCI services or the elderly customized care services, and adding deficient services (housing provision, home repair, and welfare services, such as meal assistance and moving support). The central government planned a service delivery system centered around the local government. The local governments evaluate the multiple care needs of participants at a single place within the local governments and provided integrated care services to meet their needs. In expansion to South Korea, numerous nations such as Britain, Canada, and Japan begun programs for community care among more seasoned grown-ups by joining wellbeing and social care.

Those who are eligible for the pilot project can apply to participate in the project themselves, or they can find qualified candidates at community centers or various service providers and request them to the local government. Once an application for a project is completed, the local government or community center identifies the target's complex needs. Based on the results of this needs assessment, various experts in the region gather together to form an integrated support conference on a regular basis, and the final service plan for the target is finalized through this meeting. Then, requests are made to service providers based on the service provision plan, and each service provider provides services tailored to the needs of the recipient. The local government comprehensively manages a series of service provision procedures that regularly monitor whether services are being provided to recipients according to plan.
